# Radiant Heat and Warmed Air

**Published:** 1905-06-03

**Authors:** T. J. Codd


### EDITOR'S LETTER-BOX.

[Our Correspondents are reminded that prolixity is a great bar to publication, and that brevity of style and conciseness of statement greatly facilitate early insertion.]

### RADIANT HEAT AND WARMED AIR.

*To the Editor of* The Hospital.

Dear Sir,—I beg to address you upon a question of some importance to those concerned with the design or the management of public institutions, in respect to the effect upon the human body of modern means of heating depending, broadly-speaking, upon the conveyance of heat by radiation and convection respectively. The question of open fireplaces *versus* heating by hot water or steam, etc., is an old one, but it is generally looked at more in respect of fuel consumption, convenience, and first cost, than from a standpoint having in view the essentially different physical effects of the two methods. I therefore offer the following remarks more in the manner of suggestions for discussion than as actual statements of fact.

Everyone has experienced, in a cold room with a fire in it, the sensation of being warm on the side facing the fire and chilly on the other, the reason of course being that the heat rays striking upon the body are absorbed, so raising the temperature of that part, while those not impinging upon the body pass through the air, practically without warming it, until they are received upon the walls and furniture of the room in varying degree depending upon the distance.

In heating by hot water, radiant heat is practically absent, the heat being imparted by convection alone, as shown by the fact that a sheet of dry paper might be placed within a few inches of a radiator for hours without becoming scorched, although the air in the room might become warmed to an unbearable extent. The term "radiator" is therefore really a misnomer, as has often been remarked, but the human body is itself a "radiator" in the sense in which the term is generally used; and owing to this cause, in cold climates where the truly radiant heat received from the sun is slight, the natives support the heat necessary to be maintained in their bodies to a large extent by a diet imparting heat.

It is impracticable to heat by convection to a temperature at which the body will not part with heat to the surrounding air, for to do that we should have to keep our rooms near 98° F. What, then, must be the effect upon persons living for long periods in rooms heated by this method of convection alone?

I suggest that it must at length result in a sensation of chilliness, although the room to a person who has not been in it for a considerable length of time, depending upon the state of health and constitution, may seem to be at a comfortable temperature.

I have myself experienced this inherent and unavoidable defect of heating systems depending upon heat imparted to the air only, and I have made these observations with the intention of suggesting that it is necessary in choosing a heating system for hospitals, etc., to remember that radiant heat must be resorted to in conjunction with heat by convection process if comfortable and healthy results are to be achieved.

It is, of course, feasible to heat meeting halls or offices quite satisfactorily by convection systems, for as a rule these are not tenanted by any one person for a sufficient length of time for the phenomenon I have described to be noticed, except in a few individual cases in which poor health is generally the explanation of the effect being felt sooner than usual. Yours truly,

T. J. CODD, A.M.I.M.E., M.S.E.

